# Finite element study of stem cells under fluid flow for mechanoregulation toward osteochondral cells

**DOI:** 10.1007/s10856-021-06545-3

**Published:** 2021-07-08

**Authors:** Mehdi Moradkhani, Bahman Vahidi, Bahram Ahmadian

**Affiliations:** Division of Biomedical Engineering, Department of Life Science Engineering, Faculty of New Sciences and Technologies, University of Tehran, Tehran, Iran

## Abstract

Investigating the effects of mechanical stimuli on stem cells under in vitro and in vivo conditions is a very important issue to reach better control on cellular responses like growth, proliferation, and differentiation. In this regard, studying the effects of scaffold geometry, steady, and transient fluid flow, as well as influence of different locations of the cells lodged on the scaffold on effective mechanical stimulations of the stem cells are of the main goals of this study. For this purpose, collagen-based scaffolds and implicit surfaces of the pore architecture was used. In this study, computational fluid dynamics and fluid-structure interaction method was used for the computational simulation. The results showed that the scaffold microstructure and the pore architecture had an essential effect on accessibility of the fluid to different portions of the scaffold. This leads to the optimization of shear stress and hydrodynamic pressure in different surfaces of the scaffold for better transportation of oxygen and growth factors as well as for optimized mechanoregulative responses of cell–scaffold interactions. Furthermore, the results indicated that the HP scaffold provides more optimizer surfaces to culture stem cells rather than Gyroid and IWP scaffolds. The results of exerting oscillatory fluid flow into the HP scaffold showed that the whole surface of the HP scaffold expose to the shear stress between 0.1 and 40 mPa and hydrodynamics factors on the scaffold was uniform. The results of this study could be used as an aid for experimentalists to choose optimist fluid flow conditions and suitable situation for cell culture.

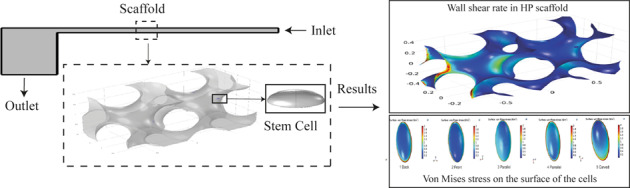

## Introduction

Today, some researchers are concerned about providing alternatives to damaged organs and tissues of patients; since it has been observed in some cases that the use of bone grafts can transmit disease and inflammation to the target area. In this regard, using of stem cells in bone scaffolds has been proposed as a strategy for tissue regeneration [1, 2]. Mesenchymal stem cells have the potential to differentiate into various target cells such as bone cells. Accordingly, these cells have wide applications in tissue engineering [3, 4].

Usually Manufacturing of controlled microstructure of scaffolds cannot be attained unless by using of the 3D-bioprinting technology [5]. By comparing the scaffolds produced via traditional methods and 3D-printed ones, it could be realized that by using additive manufacturing, scaffolds encompassing modern architectures such as implicit architecture can be built. A scaffold with implicit surfaces could modify cell culture outcomes as compared to the old method of salt leaching and also can provide a more uniform cell population in it [5, 6]. In addition, these scaffolds are capable of providing reasonable amounts of shear stress resulted from fluid flow and proper situation for nutrition and oxygenation in different regions of the scaffold. In the study conducted by Melchels et al. [7] effect of gradient porosity of the scaffold on the cells attached to the scaffold surfaces have been investigated. Results of this study showed that distribution of the cells, population in a scaffold with invariant porosity is uniform but in the gradient porosity scaffold, a variable distribution of cell population can be seen.

The effect of scaffold architecture on hydrodynamic characteristics in it has been investigated in order to provide the proper cell culture environment [8–10]. In the study of Bousti et al. [9] three groups of scaffolds were fabricated and effect of scaffold architecture on shear stress applied to the cell was investigated. The results of this study showed that the pore size and porosity affected on the shear stress distribution on different surfaces of the scaffold.

Using finite element methods in analyzing biological systems e.g., analysis of the stress and the strain induced to a cell by different stimulations proceed more than a decade. Using of 2D models can be noted among some pioneering studies in this topic including the ones that investigated the stress induced to a cell through external loadings [11, 12] and some other sophisticated 2D and 3D models in which the effect of loading on mechanical modulation of the cell have been studied [13, 14]. In some studies, cell was assumed as a rigid body (i.e., not deformable) under the induced stress from the fluid flow. Although these assumptions can approximately represent the induced shear rate on the surface of the cell body, it is better to investigate this topic by fluid-structure interaction (FSI) method through which a cell can be considered more accurately in mechanical property. As a result, this method provides us with more accurate results for the induced stresses on cells due to fluid flow [15, 16]. In the study of Zhav et al. [2] mechanoregulative response of osteoblasts in the scaffold was investigated. In this study [2], the cell was considered with nucleus and cytoplasm as its components. Computational fluid dynamics (CFD) methods were used to obtain pressure and velocity of fluid and FSI technique was used to evaluate shear stress and strain on osteoblasts.

To investigate the effect of fluid shear stress on cell growth and differentiation factors in bone scaffolds, a study was done by Eqbali et al. [17]. The results of this study showed that flow affected on the cell’s metabolic processes. In vitro observations indicated that using oscillatory fluid flow in a bioreactor can facilitate stem cell differentiation and enhance osteogenic differentiation of them [18]. In the former simulations [11, 15, 19], laminar fluid flow was considered to evaluate velocity and pressure gradients in the scaffolds.

In this study, CFD method was used to evaluate hydrodynamic factors on the surfaces of the scaffold. Besides, FSI method was used to investigate the effects of the fluid flow on the stem cell. Unlike traditional scaffolds studied by the former computational studies [19, 20], innovative aspect of this study is a comprehensive deliberation at the effect of scaffold geometry on the hydrodynamic factors of the scaffold and specifically, on shear stress induced to the stem cell. This issue is investigated for mechanical modulation study. Moreover, the effect of oscillatory fluid flow on the hydrodynamic factors in the optimized scaffold is evaluated.

## Materials and methods

### Geometry

**Fig. 1:**
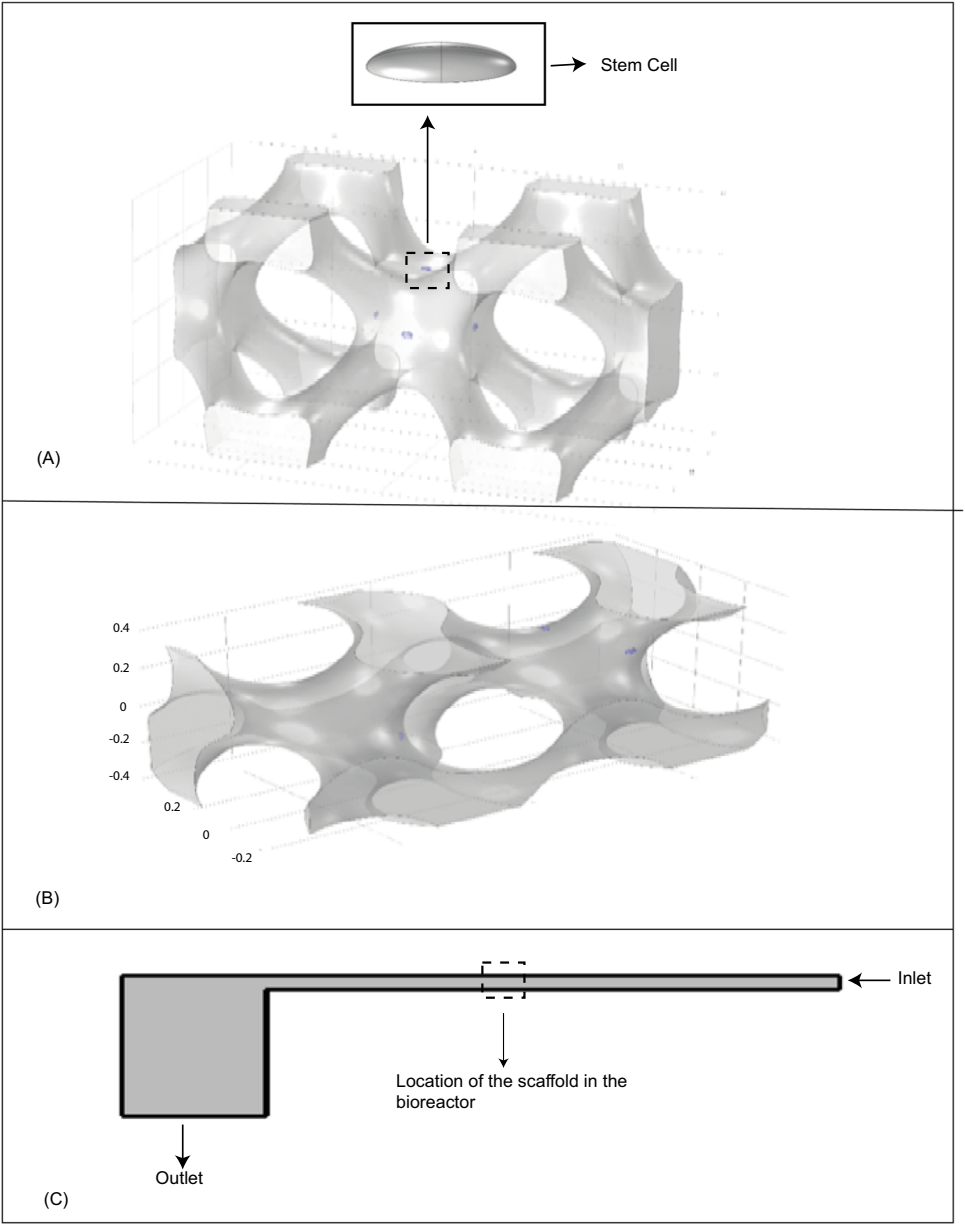
View of (**A**) IWP scaffold with cells on its surface, (**B**) HP scaffold with cells on their surface, and (**C**) the bioreactor designed for stimulation of the stem cells

The cell body has been assumed to have a perfect elliptical geometry which is inspired by real stem cell body photographed via medical imaging tools [21]. The Young modulus and Poisson’s ratio were set to 490 Pa [22] and 0.49 [23, 24], respectively. The cell is attached to a rigid substrate of the scaffold at the bottom (Fig. 1A).

Steady and unsteady fluid flow in a PPFC bioreactor has been chosen among different methods of mesenchymal stem cell stimulations for osteochondral differentiation. To do that, a sample geometry of PPFC bioreactor was considered based on a former investigation (Fig. 1C) [16]. This model of bioreactor includes an inlet for the fluid, a narrow channel between two parallel plates in which the scaffold is located with the length and width of 20 and 0.2 mm, respectively. Also, a squared outlet chamber was considered at the downstream with the length of 5 mm (Fig. 1C). The scaffold is assumed to be located in the central region of the bioreactor considering the fully developed flow condition.

In this study, a wider analysis was employed as compared to the former studies [19, 20] to evaluate the role of geometry of the scaffolds in providing optimum cell culture environment in terms of the working flow features like hydrodynamic pressure and shear stress. To do this, three different types of modern geometries for scaffolds were chosen. In order to design these scaffolds having implicit surfaces by using the trigonometry equations [6], three types of implicit scaffolds were designed in the Solid Works 2014 (Fig. 1A, B).

### The governing equations

The governing equations for a laminar incompressible flow of a Newtonian fluid are the continuity and Navier-Stokes equations as below [25]:

1 $$\nabla \cdot \left( {\rho {\boldsymbol{u}}_{fluid}} \right) \,=\, 0$$

2 $$\rho \left( {{\boldsymbol{u}}_{fluid} \cdot \nabla } \right){\boldsymbol{u}}_{fluid} \,=\, \nabla \left[ { - p{\boldsymbol{I}} \,+\, \mu \left( {\nabla {\boldsymbol{u}}_{fluid} \,+\, \left( {\nabla {\boldsymbol{u}}_{fluid}} \right)^T} \right)}\right. \\ {\left. { \,-\, \frac{2}{3}\mu \left( {\nabla \cdot {\boldsymbol{u}}_{fluid}} \right){\boldsymbol{I}}} \right]}$$

The bold terms in the equations represent tensor variables and the ${\boldsymbol{I}}$ tensor is used as the auxiliary tensor in the noted equations. In the above equations, ${\boldsymbol{u}}_{fluid}$ represents fluid velocity, $\rho$is the density of the fluid, p is fluid hydrostatic pressure, and $\mu$ is the dynamic viscosity of the fluid.

For the solid domain, the governing equations related to linear elastic behavior is given as follows [24]:

3 $$\bf \bf\sigma \,=\, C:\varepsilon$$

4 $$\bf \varepsilon \,=\, \frac{1}{2}\left[ {\left( {\nabla {\boldsymbol{u}}_{solid}} \right)^T \,+\, \nabla {\boldsymbol{u}}_{solid}} \right]$$

where $\bf \sigma$ is the Cauchy stress tensor, **C** is the stiffness coefficient tensor, $\bf \varepsilon$ is a representative for strain tensor, and ${\boldsymbol{u}}_{solid}$ is displacement tensor of the solid domain.

FSI method was used to analyze this problem which couples the interaction between fluid and solid domain at each time step [26]. The governing equations for kinematic and dynamic equilibrium at the interface of the solid and the fluid is mentioned as follows [26]:

5 $${\boldsymbol{u}}_{fluid} \,=\, {\boldsymbol{u}}_w$$

6 $${\boldsymbol{u}}_w \,=\, \frac{{\partial {\boldsymbol{u}}_{solid}}}{{\partial t}}$$

7 $$\bf \sigma \cdot {\boldsymbol{n}} \,=\, {\boldsymbol\Gamma} \cdot {\boldsymbol{n}}$$

8 $${\Gamma} \,=\, \left[ { - p{\boldsymbol{I}} \,+\, \mu \left( {\nabla {\boldsymbol{u}}_{fluid} \,+\, \left( {\nabla {\boldsymbol{u}}_{fluid}} \right)^T} \right)} \right.\left. { \,-\, \frac{2}{3}\mu \left( {\nabla \cdot {\boldsymbol{u}}_{fluid}} \right){\boldsymbol{I}}} \right]$$

where ${\boldsymbol{u}}_{fluid}$and ${\boldsymbol{u}}_w$ are the velocity tensor of fluid and velocity tensor of the wall in the interface of the fluid and the structure, respectively and Γ is the hydrodynamic stress transmitted from the fluid domain to the solid domain.

### Boundary conditions

In this study, according to the study of Zhav et al. [2] laminar flow regime was considered in the bioreactor. Afterwards, CFD analysis has been carried out regardless of the presence of the scaffold compliance to evaluate dynamic characteristics of the fluid flow in different regions of the domain. Subsequently, these results could be used as boundary conditions for the final stage analysis in which the FSI specified domain is a smaller part of the CFD domain. In this study, the fluid was considered to be water, hence the density and dynamic viscosity were considered 993 kg/m^3^ and 6.8 × 10^−4^Pa.s, respectively [27]. For inlet velocity and outlet pressure of fluid, amounts of 0.001 m/s and 4.17 Pa were set, respectively [28].

Experimental report of Chen et al. [18] showed that by using oscillatory fluid flow, differentiation of stem cells toward osteogenic fate is facilitated. Accordingly, in the final step of this study, effects of oscillatory fluid flow on the hydrodynamic factors in the optimized scaffold were investigated. At the inlet of the bioreactor assuming oscillatory flow condition, mean chamber velocity equals to 30 + 300πsin(2πt) μm/s was specified and a constant pressure magnitude equals to zero was assigned at the outlet [18]. Finally, Effect of frequency of the oscillatory fluid flow on shear stress distribution on the surface of the scaffold was evaluated in two periods.

### Solution method

In this study, CFD method was used to evaluate the role of fluid flow’s regional characteristics as well as the effect of stem cell, location in the scaffold on its fate. This analysis was performed at the first step to determine the convenient surfaces to locate the stem cells. In this localization process, three effective factors were considered as follows:

1. The shear rate on the scaffold surfaces due to the fluid flow,
2. The fluid pressure distribution in the scaffold.
3. The direction of surface normal vector relative to the fluid flow which should be in four directions of parallel, contrary, oblique and perpendicular arrangements with respect to initial velocity vectors of flow at the inlet boundary.

At this step, according to the results achieved from the CFD model, the stem cell was located on the chosen surface of the scaffold. According to Fig. 1C, the fluid domain used in this simulation was identified by the specified box. This domain is assumed to be a portion of whole PPFC bioreactor and the scaffold was placed in the middle of this fluid domain (Fig. 1C). In fact, this small domain is chosen out of the real large domain of fluid to reduce the computational cost of the simulation. However, the initial and boundary conditions such as inlet velocity and outlet pressure for the FSI domain is adapted from the CFD analysis of fluid flow in the PPFC bioreactor. The lateral faces of the FSI box is considered to have symmetry because of the repeatable pattern of the scaffold geometry to build a complete practical scaffold in reality. The normal velocity of fluid was set to zero in these faces to avoid any fluid flow inward and outward of the faces.

In this study, COMSOL Multiphysics 5.2 (PaloAlto, CA) was used. The numerical solution was carried out based on the finite element method. Meshing quality for different domains is reported in Table 1. Due to significant size differences between the cell and the scaffold, there should be a fine quality of meshing at the interface of the cell and its neighboring domains in order to accurately transfer data at these regions. In addition, the prism element has been selected for the boundary layer of fluid flow because of its precision in high gradient shear rate evaluations in comparison with quadrilateral element which was used for the rest domain of fluid flow. Furthermore, triangle elements were used for the surface of the scaffold.

**Table 1:** Number of elements used in this study

Type of meshes of the scaffold	Fluid domain		Scaffold wall	Cells	Whole geometry
	Prism	Tetrahedral	Triangular	Tetrahedral	
IWP	62,264	341,753	30,554	27,210	431,227
Gyroid	67,710	293,284	23,179	42,056	403,050
HP	58,994	359,133	24,088	31,153	449,310

To study mesh independency of the solution, the number of elements was increased for two times and shear stress applied to the cell surface were investigated. As a result, the maximum differences in these parameters in most points were lower than 3%. For this reason, the number of elements used for the analysis was shown in Table 1.

## Results

### Effect of scaffold architecture on wall shear rate and flow streamlines

**Fig. 2:**
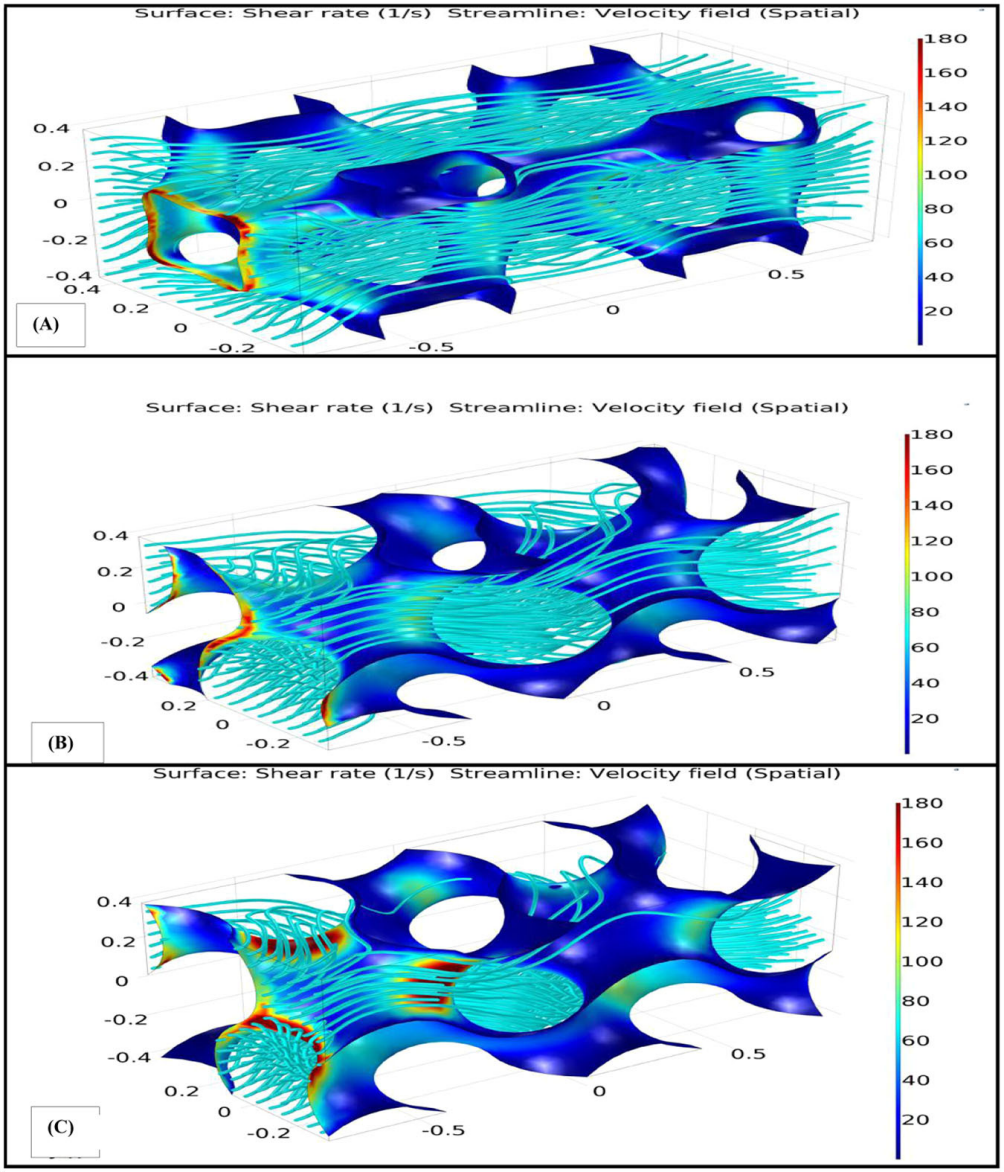
Shear rate and flow streamlines for (**A**) IWP scaffold, (**B**) HP scaffold, and (**C**) Gyroid scaffold. This result has been achieved with inlet velocity of 0.001 m/s and outlet pressure of 4.17 P

To distinguish fluid flow hydrodynamic characteristics near the faces of the scaffolds on which the stem cells are normally lodged, values of wall shear rate and hydrodynamic pressure in the scaffold have been obtained. These results provide an opportunity to find the reasonable areas in a scaffold that could offer optimum microenvironment for the cultured cells and also may lead to control the hydrodynamic characteristics that could affect the results of an in vitro experiment. Wall shear rate and flow streamlines were obtained from the CFD analysis of the Gyroid, HP and IWP scaffolds that can be seen in Fig. 2. As shown in this figure, in the area where number of streamlines was enhanced, amount of shear rate was increased remarkably and maximum shear stress occurred in this region.

### Effects of scaffold’s geometry on stresses applied to the cell

One of the main goals of the current study is to investigate the effect of scaffold architecture on the transmitted stresses to the cultured cells in different portions of the scaffold. Accordingly, the response of the cells in terms of stress and deformation were analyzed. The results for each scaffold are discussed as follows.

In the IWP scaffold, the complexity of the surfaces was less than the other scaffolds used for the simulation. In this regard, the selected spots for locating stem cells were not very diverse. Location of the cells on the scaffold divided in three groups as noted below:

1. Two cells were located faced to the fluid flow.
2. Two cells were assumed to be located parallel with the fluid flow direction.
3. Two cells were assumed to be located on the diagonal surfaces of the scaffold where the amount of share rate is relatively higher than other surfaces of the scaffold.

**Fig. 3:**
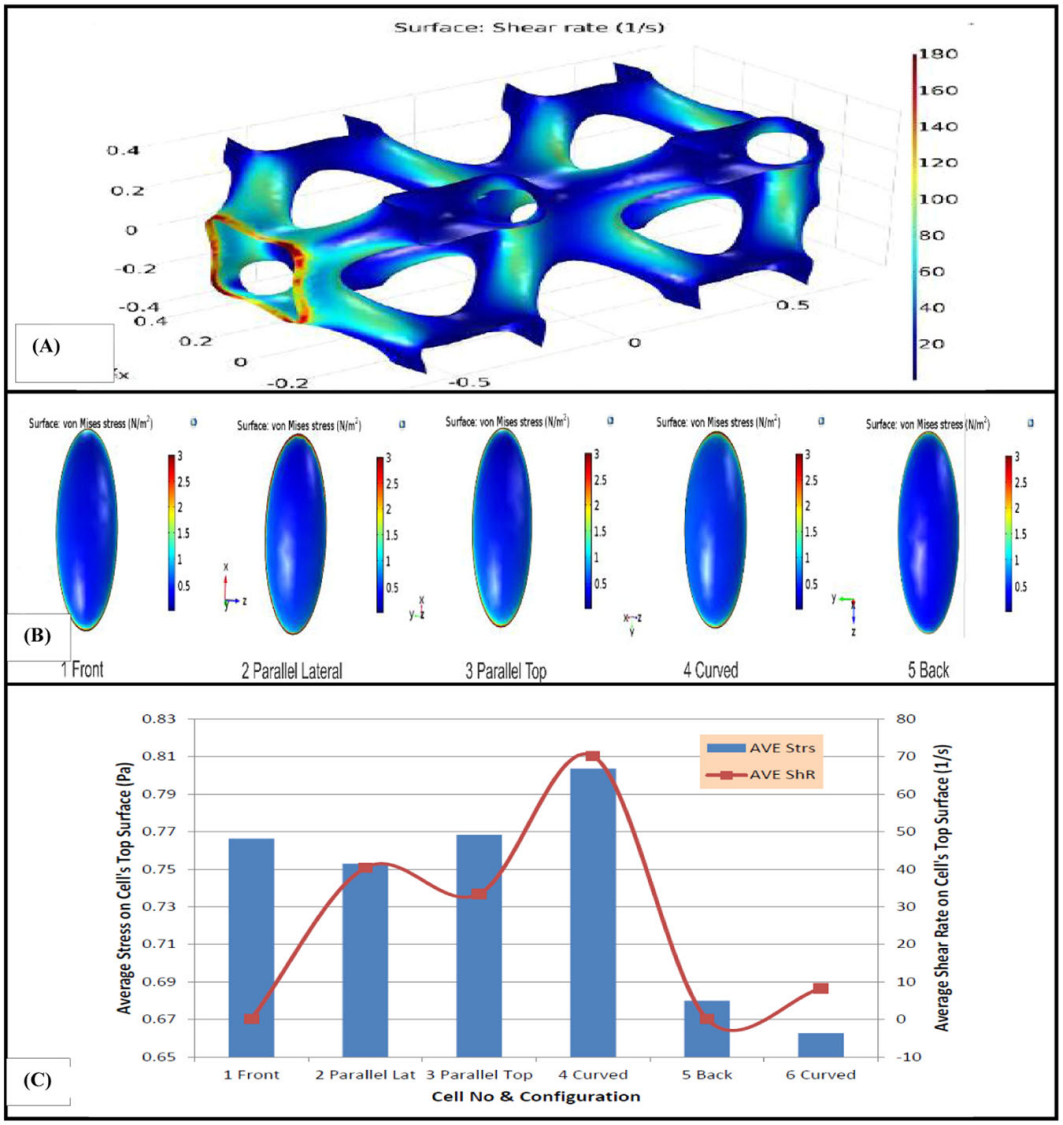
**A** Shear rate in different parts of IWP scaffold, (**B**) von Mises stress in the top surface of the cells placed in different spots of the scaffold. **C** Average shear rate and average amount of shear stress and von Mises stress in the top surface of the inhabited cells in different spots of IWP scaffold

The resulted shear rate on the stem cell in IWP scaffold as illustrated in Fig. 3, shows that the cell located faced to fluid flow exposed to minimum amount of shear rate compared to the other cells. In addition, maximum shear rate which is ~180/s occurred in the entrance of scaffold due to the small cross section of the inlet. Figure 3C provides a comparative analysis of average amount of shear stress and von Mises stress on the top of the cell. As shown in Fig. 3C, maximum and minimum of average amount of shear stress and von Mises stress experienced by the cells are 0.8 and 0.66 Pa, respectively. These maximum and minimum was occurred on the cell which was located on the diagonal surfaces of the scaffold.

**Fig. 4:**
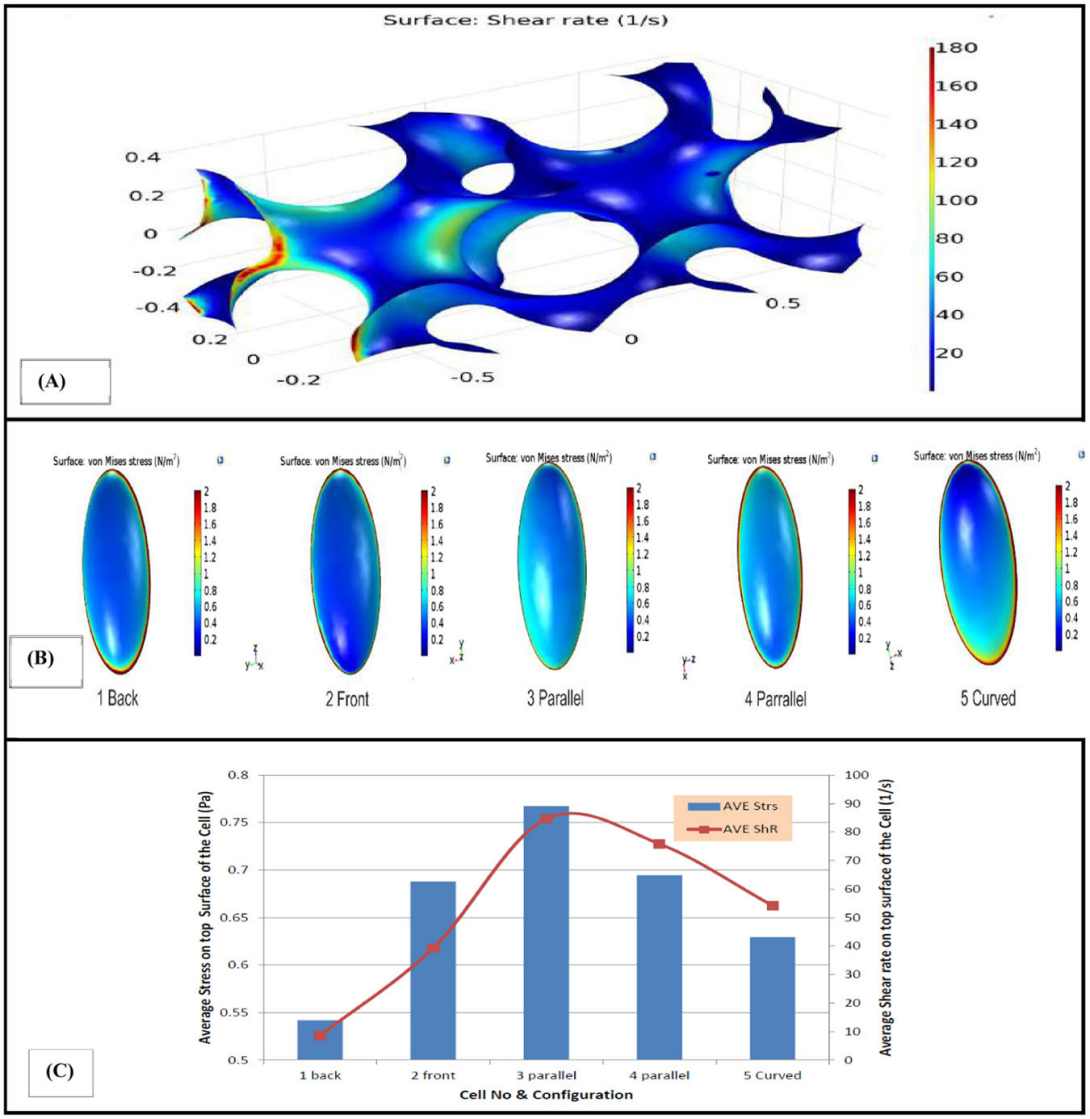
**A** Wall shear rate in different parts of HP scaffold, (**B**) von Mises stress in the top surface of the cells placed in different spots of the scaffold. **C** Average shear rate and average amount of shear stress and von Mises stress in the top surface of the inhabited cells in different spots of HP scaffold

For this scaffold, five stem cells were located in different regions exposing to different amounts of shear rate. It should be noted that a significant variation of hydrodynamic pressure was not observed in this type of scaffold. Figure 4 presents the wall shear rate graph in different portions of the scaffold and von Mises stress on the surface of each cell in different locations. In addition, average amount of shear stress and von Mises stress experienced by the top surface of each cell and the amount of average shear rate of fluid flow at that surface can be seen in Fig. 4C.

**Fig. 5:**
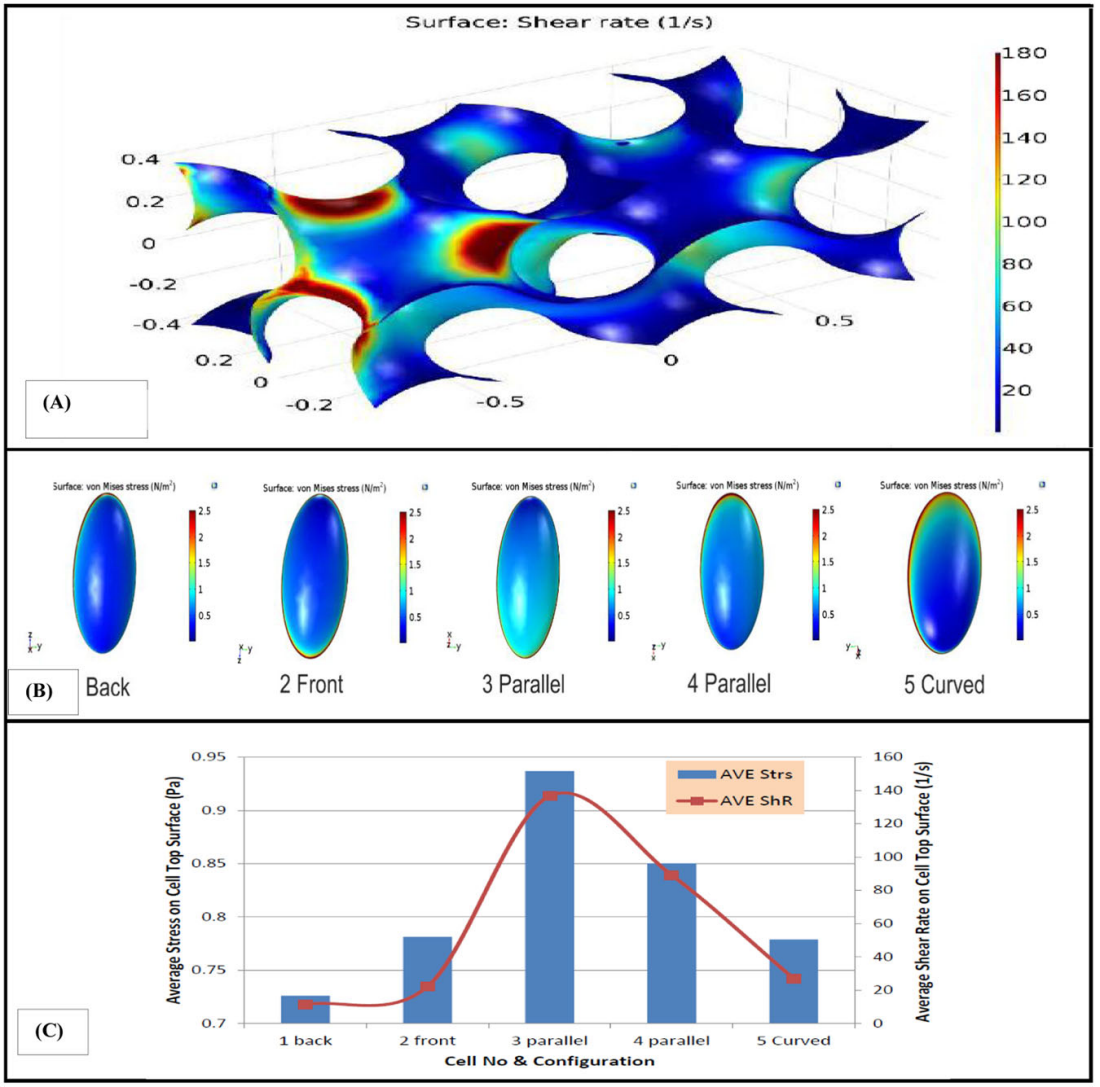
(**A**) Wall shear rate in different parts of Gyroid scaffold, (**B**) von Mises stress in the top surface of the cells placed in different spots of the Gyroid scaffold. (**C**) Average shear rate and average amount of shear stress and von Mises stress in the top surface of the inhabited cells in different spots of Gyroid scaffold

The third scaffold which is used in this study has Gyroid architecture. According to the CFD results, five cells were located in the first 30 percent of this scaffold. These five cells were placed on the surfaces with different shear rate ranges. von Mises stress in the top surface of the cell can be observed in Fig. 5B. As shown in Fig. 5B, maximum von Mises stress was occurred in the substrate of the cell. The average amount of shear stress and von Mises stress in the top surface of the cell and also the fluid’s shear rate on that surface for different cells placed on different surfaces of this scaffold can be observed in Fig. 5C. It can be observed that maximum shear rate and average stress occur in the parallel cell. Moreover, shear stress in this type of scaffold is higher than other types of presented scaffolds.

### Effect of oscillatory fluid flow on shear rate distribution

**Fig. 6:**
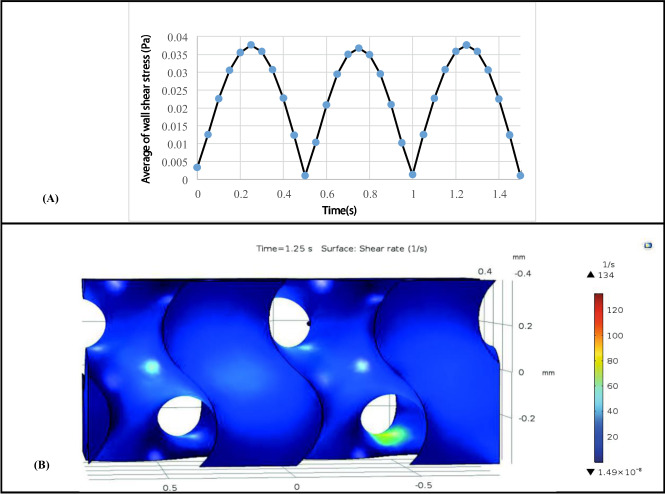
**A** Average of wall shear stress on the whole surface of Hp scaffold during two periods, (**B**) Wall shear rate distribution on different surfaces of the HP scaffold under oscillatory fluid flow in *t* = 1.25 s

Effect of oscillatory fluid flow on the top surface of HP scaffold was shown in Fig. 6. The results illustrated that effect of time period on the average of shear stress magnitude is infinitesimal (Fig. 6A). It can be seen the maximum Magnitude of wall shear stress occurred on the surface of the scaffold is approximately 0.036 Pa in *t* = 1.25 s and the minimum wall shear stress occurred on the surface of the scaffold is approximately 0.001 Pa in *t* = 0.5 s. Effect of oscillatory fluid flow on shear rate distribution on the surface of HP scaffold was shown in Fig. 6B. As it can be concluded, shear rate distribution on the surface of this scaffold was uniform and most surfaces of the scaffold were exposed to the same shear rate about 25 1/s.

## Discussion

In this investigation, three different architectures of scaffolds with implicit surface were used to investigate mechanical modulation of stem cells cultured on the scaffold under fluid flow stimulations. These types of scaffolds are among the modern architectures used in tissue engineering [5, 6] and to the best of our knowledge, they have never been investigated alongside with the cultured stem cells by the means of numerical methods and specifically using FSI method.

The results of the CFD analysis showed that based on the flow streamlines, oxygen and nutrition delivery to different portions of the Gyroid scaffold is very similar to the HP scaffold. From the mathematical equations for Gyroid and HP scaffold, it can be concluded that Gyroid and HP architectures are very analogous to each other in terms of the equation [6], unless Gyroid scaffold has less porosity than the HP scaffold. In addition, slow velocity in the Gyroid scaffold is more prevalent than the HP scaffold due to decreasing in the cross-sectional area of the channels specifically at the entering section of fluid flow and occurrence of non-developed flow region. So, neighboring to this surface is not a suitable location for cells to be lodged.

Some studies showed that variant distribution of wall shear stress provide nonuniform cell culture in the scaffolds [29, 30]. So, investigating the distribution of shear rate on the surface of the scaffolds seems to be important for mechanical modulation of stem cells. Hence, in this study distribution of shear rate on the surface of different scaffolds was evaluated. The results of shear rate on the stem cell in the IWP scaffold showed that the cell located faced to the fluid flow was exposed to minimum amount of shear rate as compared to the other cells. It is mainly because of low amount of velocity magnitude of fluid flow near this surface. As a result, stem cells should not be lodged there, because of the negligible amount of shear rate and velocity of fluid on the scaffold. Magnitude of shear rate on some surfaces was about 1000 times higher than dead surfaces as it can be seen in Fig. 2. This result showed that achievable surfaces to culture stem cell in this type of scaffold are too small. Regardless of shear rate amount in different portions of the scaffolds, the average amount of shear stress and von Mises stress induced to stem cells located on the scaffolds in this simulation varies between 0.55 and 0.9 Pa which properly meets the minimum amount of desired stresses to culture stem cells in a bioreactor [31].

By comparing the wall shear rate in the three different types of scaffolds, it is simply comprehended that the HP scaffold provides more optimized surfaces to culture stem cells. Although high shear rate of fluid flow was observed at the entry region of the scaffold, the last three quarters long of the scaffold, constant amount of shear rate from fluid flow at different regions of the scaffold surface was observed. One reason for this smoothness is that the architecture of this scaffold helps the fluid to flow almost with a constant and similar velocity in different channels of the scaffold. This is very important in delivery of desired amount of nutrition and oxygen to all portions of the scaffold equally and providing the controllable and suitable quality of hydrodynamic pressure and shear rate from fluid flow for mechanoregulation of a specific cell in that scaffold [32]. This result predicts that if other biological conditions such as chemical environment are designed properly, by adjusting inlet velocity and outlet pressure in the bioreactor, the growth and differentiation of stem cells placed in various locations of this type of scaffold can be modulated.

Experimental results showed that oscillatory fluid flow could provide a higher survival surface for stem cells [18]. In this regard, effect of oscillatory fluid flow on the mesenchymal stem cells in the optimized scaffold (HP scaffold) was investigated. The result of shear rate distribution on the surface of this scaffold due to oscillatory fluid flow showed that hydrodynamic factors in the HP scaffold was uniform and most surfaces of the scaffold was suitable for cell culture. This result is in accordance with the reports of Chen et al. [18].

One of the limitations of this research is computational costs of modeling. As a consequence, some simplifications such as in mechanical properties of the cells were assumed. To continue this research, it is suggested to investigate effect of pore size of implicit scaffolds and distribution of pores in the scaffold on stress transmission to the cells. Furthermore, by using confocal microscopy, cell geometries can be assumed more accurately and modeling of cells assuming hyperplastic and viscoelastic properties may be useful to give exact prediction of cell response.

## Conclusion

In this research, effect of scaffold architecture due to different implicit surfaces on the hydrodynamics factors of the scaffold as well as shear stress induced to the attached stem cell have been investigated. The results show that the HP scaffold provides more optimized surfaces to locate stem cells rather than other types of the scaffolds studied in this research. Moreover, the result of exerting oscillatory fluid flow to the HP scaffold indicated that dead zones of the scaffold, where is not suitable for cell seeding, was decreased. Although the parameters affecting on modulations of a stem cell are numerous, most of them have complex natures and much of their functional mechanisms are not completely recognized. The results achieved in these simulations may be beneficial for better understanding of the interaction between culture medium fluid, cell and scaffold and also environmental conditions of the cell in the micro scale. Therefore, before building a specific scaffold, its design can be optimized and during the test, values of fluid flow parameters such as inlet velocity and outlet pressure can be adjusted in a way that the cells being seeded, would be in the most optimized condition in the laboratory environment.
